# Endogenous T1ρ cardiovascular magnetic resonance in hypertrophic cardiomyopathy

**DOI:** 10.1186/s12968-021-00813-5

**Published:** 2021-10-25

**Authors:** Elizabeth W. Thompson, Srikant Kamesh Iyer, Michael P. Solomon, Zhaohuan Li, Qiang Zhang, Stefan Piechnik, Konrad Werys, Sophia Swago, Brianna F. Moon, Zachary B. Rodgers, Anya Hall, Rishabh Kumar, Nosheen Reza, Jessica Kim, Alisha Jamil, Benoit Desjardins, Harold Litt, Anjali Owens, Walter R. T. Witschey, Yuchi Han

**Affiliations:** 1 Department of Bioengineering, School of Engineering and Applied Science, University of Pennsylvania, Philadelphia, PA USA; 2 Perelman School of Medicine, University of Pennsylvania, Philadelphia, PA USA; 3 Department of Radiology, University of Pennsylvania, Philadelphia, PA USA; 4 Division of Cardiovascular Medicine, Department of Medicine, University of Pennsylvania, Philadelphia, PA USA; 5 Ultrasound in Cardiac Electrophysiology and Biomechanics Key Laboratory of Sichuan Province, Cardiovascular Ultrasound and Non-Invasive Cardiology Department, Affiliated Hospital of University of Electronic Science and Technology of China, Sichuan Academy of Medical Sciences, Sichuan Provincial People’s Hospital, Chengdu, Sichuan China; 6 Oxford Center for Clinical Magnetic Resonance Research, Oxford BRC NIHR, Division of Cardiovascular Medicine, Radcliffe Department of Medicine, University of Oxford, Oxford, UK; 7 Circle Cardiovascular Imaging Inc., Calgary, AB Canada; 8 Department of Biophysics, University of Pennsylvania, Philadelphia, PA USA; 9 Department of Medicine, University of Pennsylvania, Philadelphia, PA USA; 10 Perelman School of Medicine, University of Pennsylvania, 11-135, South Pavilion, 3400 Civic Center Blvd., Philadelphia, PA 19104 USA

**Keywords:** Hypertrophic cardiomyopathy, T1ρ, LGE, T1

## Abstract

**Background:**

Hypertrophic cardiomyopathy (HCM) is characterized by increased left ventricular wall thickness, cardiomyocyte hypertrophy, and fibrosis. Adverse cardiac risk characterization has been performed using late gadolinium enhancement (LGE), native T1, and extracellular volume (ECV). Relaxation time constants are affected by background field inhomogeneity. T1ρ utilizes a spin-lock pulse to decrease the effect of unwanted relaxation. The objective of this study was to study T1ρ as compared to T1, ECV, and LGE in HCM patients.

**Methods:**

HCM patients were recruited as part of the Novel Markers of Prognosis in Hypertrophic Cardiomyopathy study, and healthy controls were matched for comparison. In addition to cardiac functional imaging, subjects underwent T1 and T1ρ cardiovascular magnetic resonance imaging at short-axis positions at 1.5T. Subjects received gadolinium and underwent LGE imaging 15–20 min after injection covering the entire heart. Corresponding basal and mid short axis LGE slices were selected for comparison with T1 and T1ρ. Full-width half-maximum thresholding was used to determine the percent enhancement area in each LGE-positive slice by LGE, T1, and T1ρ. Two clinicians independently reviewed LGE images for presence or absence of enhancement. If in agreement, the image was labeled positive (LGE + +) or negative (LGE −−); otherwise, the image was labeled equivocal (LGE + −).

**Results:**

In 40 HCM patients and 10 controls, T1 percent enhancement area (Spearman’s rho = 0.61, p < 1e-5) and T1ρ percent enhancement area (Spearman’s rho = 0.48, p < 0.001e-3) correlated with LGE percent enhancement area. T1 and T1ρ percent enhancement areas were also correlated (Spearman’s rho = 0.28, p = 0.047). For both T1 and T1ρ, HCM patients demonstrated significantly longer relaxation times compared to controls in each LGE category (p < 0.001 for all). HCM patients also showed significantly higher ECV compared to controls in each LGE category (p < 0.01 for all), and LGE −− slices had lower ECV than LGE + + (p = 0.01).

**Conclusions:**

Hyperenhancement areas as measured by T1ρ and LGE are moderately correlated. T1, T1ρ, and ECV were elevated in HCM patients compared to controls, irrespective of the presence of LGE. These findings warrant additional studies to investigate the prognostic utility of T1ρ imaging in the evaluation of HCM patients.

## Background

Hypertrophic cardiomyopathy (HCM), characterized by an unexplained increase in left ventricular (LV) wall thickness, is the most common genetic cardiac disorder, with a prevalence of approximately 1 in 500; this prevalence may be as high as 1 in 200 when accounting for both genotype-positive/phenotype-positive and genotype-negative/phenotype-positive individuals [1]. Typical pathologic findings of HCM include cardiomyocyte hypertrophy and disarray, as well as focal or diffuse interstitial fibrosis [2]. In recent years, cardiovascular magnetic resonance (CMR) has been used to characterize and quantify myocardial fibrosis. Increased fibrosis, seen as late gadolinium enhancement (LGE), has been identified as a risk factor for sudden cardiac death and heart failure in this population [3]. T1 mapping and extracellular volume (ECV) quantification through CMR have also been correlated with increased risk of cardiovascular events [4, 5]. However, not all HCM patients will go on to have an event; LGE has a high prevalence (as high as 70%) in this population [6, 7] but a low specificity for the prediction of future cardiovascular events, limiting its negative predictive value [8]. Additionally, gadolinium-based contrast agents (GBCAs) confer a risk of nephrogenic systemic fibrosis in patients with renal disease, and additionally are deposited in brain tissue [9, 10]. Accordingly, there is interest in the development and validation of more specific and non-contrast methods for myocardial characterization in HCM patients.

T1ρ CMR is an endogenous contrast method for tissue characterization that does not require GBCAs and is distinct from both T1 and T2 contrast. It utilizes a low power radiofrequency pulse, also called a spin-lock pulse, to enable measurement of longitudinal relaxation in the rotating frame (T1ρ). The spin lock pulse mitigates the loss of transverse magnetization, suppressing contributions to relaxation from chemical exchange and water diffusion through magnetic field gradients [11]. Its ability to detect myocardial fibrosis has been validated in animal models of ischemia and reperfusion [12–14] as well as in explanted hearts from patients with dilated cardiomyopathy [15]. Despite its mechanistic relevance to HCM pathophysiology, few studies have investigated the value of T1ρ in this population. Thus, we sought to evaluate and characterize the role of T1ρ in HCM patients by comparing it to conventional LGE and native T1.

## Methods

### Study population

We prospectively enrolled HCM patients between August 10, 2015 and July 10, 2017 as part of the Novel Markers of Prognosis in Hypertrophic Cardiomyopathy (HCMR) study. Detailed trial inclusion and exclusion criteria have been previously published [16]. In brief, key inclusion criteria were patients aged 18–65 years with an established HCM diagnosis defined as unexplained myocardial hypertrophy of ≥ 15 mm without cavity dilation, etiologies such as hypertension and aortic stenosis, or other infiltrative cardiomyopathies such as amyloidosis and sarcoidosis. Additional exclusion criteria were: (1) prior septal myectomy or alcohol septal ablation, (2) prior myocardial infarction or coronary artery disease, (3) incessant ventricular arrhythmias, (4) inability to lie flat, (5) contraindications to CMR including pacemakers, defibrillators, intraocular metal, certain types of intracranial aneurysm clips, severe claustrophobia, and stage IV/V chronic kidney disease with estimated glomerular filtration rate < 30 mL/min/1.73 m^2^, (6) diabetes mellitus with end organ damage, (7) pregnancy, and (8) inability to provide informed consent. In addition, we recruited 10 healthy subjects without cardiovascular risk factors or diseases and on no medications to serve as a control group. The study protocol was approved by the Institutional Review Board of the University of Pennsylvania and all subjects gave written informed consent prior to enrollment.

### CMR imaging

CMR was performed using a 1.5 T CMR scanner (Avanto; Siemens Healthineers; Erlangen, Germany), equipped with 18 channel anterior and posterior array coils. Retrospectively gated, short axis, multi-slice cine CMR was performed with a temporal resolution = 34–40 ms, flip angle = 70°, bandwidth = 940 Hz/pixel, spatial resolution = 1.8 × 1.8 mm^2^, slice thickness = 8 mm.

2D T1ρ breath-held single-shot balanced steady-state free precession (bSSFP) sequences were performed at 3 short axis slice positions for HCM patients (apical, mid, and basal) in systole and 2 short axis slice positions for controls (mid and basal) using a motion- and heart rate-corrected spin echo, spin lock (SL) T1ρ pulse cluster (90_x_—SL_y_—180_y_—SL_-y_—90_-x_) at end-systole [17–19]. T1ρ images were acquired with different SL times (TSL) using the following parameters: TSL = 2, 10, 18, 26, 34, 42, 50 ms, B_1_ = 400–500 Hz, spatial resolution = 1.4 × 1.4 mm^2^, slice thickness = 8 mm, flip angle = 70°, echo time (TE) = 1.45 ms, repetition time (TR) = 2.9 ms, number of segments (N_Seg_) = 55, bandwidth = 900 Hz/pixel, linear k-space phase encoding ordering, parallel imaging with acceleration factor = 2, 34 reference k-space lines obtained in a separate heartbeat, and allowing 1 additional heartbeat for T1 relaxation between shots. The T1ρ pulse amplitude was set at the highest available within scanner specific absorption rate (SAR) limits (B_1_ = 400–500 Hz). Motion correction was used to reduce residual cardiac and respiratory motion between T1ρ images (Equation [1]). The relaxation rate $R1\rho =\frac{1}{T1\rho }$ and intercept $B$ were estimated by two-parameter fit

1 $$\min _{{R1\rho ,B}} \left\| {\ln (S_{i} ) - B + R1\rho \cdot TSL_{i}} \right\|_{{2}}^{2}$$

where ${S}_{i}$ is the magnitude signal at each spin lock duration ${TSL}_{i}$. Motion correction and parametric mapping (Eq [1]) were implemented using custom C + + software on the CMR scanner [17].

2D T1 images were obtained with a breath-held shortened modified Look-Locker inversion recovery (ShMOLLI) [20] sequence at 3 short axis slice positions matched to T1ρ at mid-end-diastole [21]. Other parameters were: spatial resolution = 1.4 × 1.4 mm^2^, slice thickness = 8 mm, flip angle = 35°, TE = 1.2, TR = 2.4 ms, N_Seg_ = 57, bandwidth = 1080 Hz/pixel, linear k-space encoding, parallel imaging acceleration factor = 2, 34 reference k-space lines obtained in a separate heartbeat. These images were prospectively electrocardiogram gated.

A 0.15 mmol/kg intravenous injection of gadolinium-based contrast was used for LGE imaging (Magnevist; Bayer Schering Pharma; Leverkusen, Germany). Imaging was performed 15–20 min after injection of contrast agent using an inversion time (TI) scout sequence to determine the TI to null myocardial tissue signal. LGE CMR was obtained using a 2D segmented phase-sensitive inversion recovery (PSIR) sequence at spatial resolution = 1.2 × 1.2 mm^2^, flip angle = 50°, TE = 1.6 ms, TR = 3.2 ms, slice thickness = 8 mm, and parallel imaging acceleration factor = 2 [22].

### Image analysis

#### Cardiac function

Cardiac volumes and functional data were analyzed on the short-axis cine images using a commercially available software (Suiteheart, Neosoft, Pewaukee, Wisconsin, USA) The endocardium and epicardium were automatically traced at end-diastole and end-systole and manually adjusted following Society for Cardiovascular Magnetic Resonance guidelines [23]. Papillary muscles were included in the ventricular volume.

#### Presence of enhancement on LGE

All LGE images were anonymized, shuffled, and presented to 2 blinded expert readers (B.D. and H.L., each with > 10 years of CMR experience), who labeled each slice as showing positive visible enhancement or not. Slices were labeled as showing positive (++) or negative enhancement (–) if both experts agreed, and otherwise were labeled equivocal (+ −).

#### Determination of myocardial relaxation times, scar size, and ECV

Relaxation times were measured in pre-contrast T1, post-contrast T1, and T1ρ images by manual contouring of the LV myocardium using QMass (Medis, Leiden, Netherlands). In LGE, T1, and T1ρ images, enhancement area was quantified using full width at half maximum (FWHM) thresholding and reported as the ratio of enhanced to total LV area (%). ECV was calculated per Equation [2] using blood and entire myocardial T1 values, and hematocrit (Hct) obtained within 24 h of CMR [24].

2 $$ECV = 100\% \times \left( {1 - Hct} \right) \times \frac{{1/Myocardial\,T1_{post - contrast} - 1/Myocardial\,T1_{pre - contrast} }}{{1/Blood\,T1_{post - contrast} - 1/Blood\,T1_{pre - contrast} }}$$

### Statistical analysis

Statistical analysis was performed using R 3.6.1 (R Foundation for Statistical Computing, Vienna, Austria) and MATLAB R2019b (The MathWorks Inc., Natick, Massachusetts, USA). Categorical variables are expressed as N (%); continuous variables are expressed as mean ± SD or median [interquartile range (IQR)] depending on the distribution of the data. Normality testing was performed using the Shapiro–Wilk test. If the data were normally distributed, parametric methods were used, otherwise non-parametric methods were used. Student’s t-test, Wilcoxon Signed Rank test, one-way analysis of variance (ANOVA), and Kruskal–Wallis test (with post-hoc Dunn test adjusted with the Benjamini–Hochberg method) were used as appropriate based upon the variables and data distribution. To compare proportions of categorical variables, Chi-square test and Fisher’s exact test were used, as appropriate. The correlation between T1ρ and other parameters was assessed using Pearson’s and Spearman’s correlation coefficients, as appropriate. p values less than 0.05 were considered statistically significant.

## Results

### Patient characteristics

**Fig. 1:**
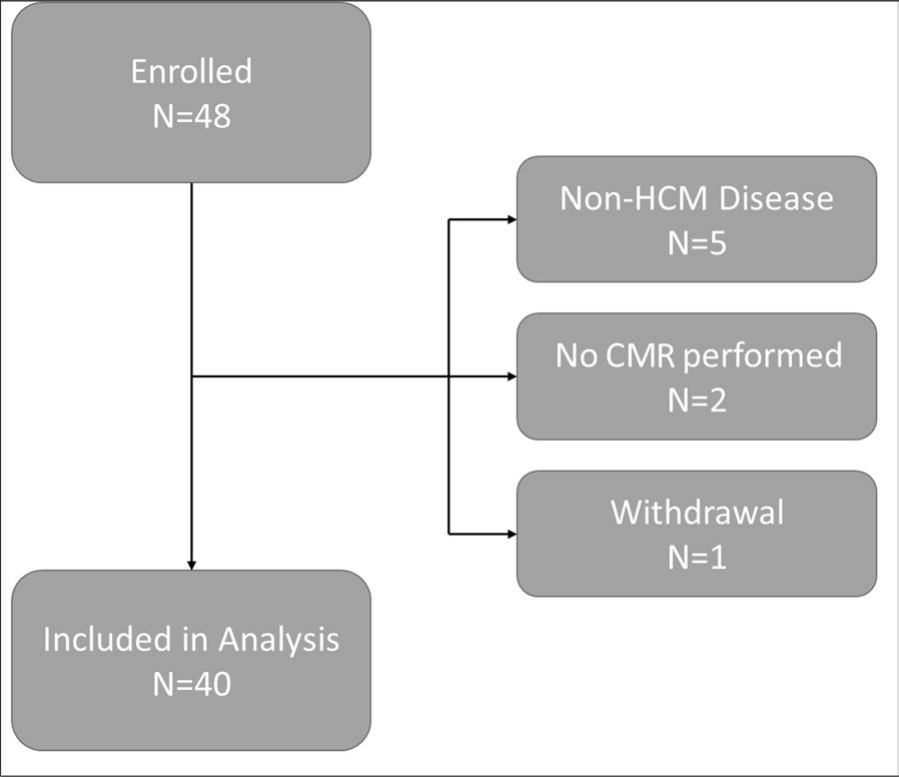
Study participant flow diagram. Disposition of hypertrophic cardiomyopathy (HCM) patients is shown; 48 subjects were enrolled, and after applying exclusionary criteria, 40 subjects were included in final analysis. *HCM* hypertrophic cardiomyopathy, *CMR* cardiovascular magnetic resonance

A total of 48 subjects were enrolled through the HCMR study [16]; 8 subjects were excluded for (1) having other diseases (n = 5), (2) no CMR performed (n = 2), and (3) withdrawal from the study (n = 1; Fig. 1). Baseline characteristics are presented in Table 1. Median age was 50 [IQR 35–57] years, 48% of patients were female, and median body surface area (BSA) was 2.0 m^2^. Controls had similar distributions of age, gender, and BSA. 30% of patients had a history of ventricular arrhythmia, and 15% had a history of syncope. Maximum LV wall thickness was 17.5 ± 3.3 mm. 35% of patients had obstruction seen on echocardiogram, and the majority had mild mitral regurgitation. 32.5% of patients had NYHA Class II heart failure, 20% of patients had Class III heart failure, and no patients had Class IV heart failure. 25% of patients had a likely pathogenic or pathogenic genetic variant.

**Table 1:** Characteristics of the HCM Patient and Control Cohorts

	HCM patients (N = 40)	Healthy Controls (N = 10)	p-value
Age (years)	50 [35, 57]	51 [38, 55]	1
Gender			0.724
Male	21 (52.5%)	4 (40.0%)	
Female	19 (47.5%)	6 (60.0%)	
BSA (m^2^)	2.0 [1.8, 2.2]	1.8 [1.7, 2.1]	0.254
Hematocrit (%)	42.0 [39, 43.3]	39.5 [37.0, 41.0]	0.002
Medical history			
Coronary artery disease	0 (0%)		
Hypertension	16 (40.0%)		
Diabetes mellitus	2 (5.0%)		
Stroke or transient ischemic attack	0 (0%)		
Hospitalization for heart failure	1 (2.5%)		
Ventricular arrhythmia	12 (30.0%)		
Syncope	6 (15.0%)		
Maximum LV wall thickness (mm)	17.5 (3.25)		
LVOT obstruction	14 (35.0%)		
Mitral regurgitation			
None	5 (12.5%)		
Mild/trace	27 (67.5%)		
Moderate	5 (12.5%)		
Severe	3 (7.5%)		
NYHA heart failure classification			
I	19 (47.5%)		
II	13 (32.5%)		
III	8 (20.0%)		
IV	0 (0%)		
ESC risk score (%)	2.19 (0.924)		
Genotype positive	10 (25.0%)		

Values are presented as Mean (Standard Deviation), Median [Interquartile Range], or N (%) depending on the distribution of the data

*BSA* body surface area, *ESC* European Society of Cardiology, *LV* left ventricle, *LVOT* left ventricular outflow tract, *NYHA* New York Heart Association

### CMR measurements and LGE ratings

CMR measurements for both HCM patients and controls are shown in Table 2. Compared to controls, HCM patients had higher LV mass (148 g vs. 94 g, p < 0.001), LV mass index (74.8 vs. 48.6 g/m^2^, p < 0.001), and LV ejection fraction (LVEF) (65.0% vs. 60.2%; p < 0.001). In the right ventricle (RV), HCM patients had lower indexed end diastolic volume (EDVI; 74.4 mL/m^2^ vs. 93.4 mL/m^2^, p = 0.011), end systolic volume (ESV; 52.6 mL vs. 81.4 mL; p = 0.001) and indexed RV ESV (RVESVI; 26.7 mL/m^2^ vs. 42.7 mL/m^2^; p = 0.001). HCM patients had higher RV ejection fraction (64.4% vs. 54.1%; p < 0.001).

**Table 2:** CMR imaging findings

	HCM patients (N = 40)	Controls (N = 10)	p-value
Left ventricle (LV)			
LV mass (g)	148 (51)	94 (32)	**< 0.001**
LV mass index (g/m^2^)	74.8 (22.8)	48.6 (11.8)	**< 0.001**
LVEDV (mL)	167 (36.0)	163 (46.5)	0.797
LVEDVI (mL/m^2^)	85.1 (13.6)	85.3 (15.8)	0.961
LVESV (mL)	58.8 (17.2)	66.1 (20.6)	0.322
LVESVI (mL/m^2^)	30.0 (7.90)	34.6 (7.73)	0.114
LV stroke volume (mL)	109 (25.3)	97.8 (26.5)	0.263
LVEF (%)	65.0 (6.18)	60.2 (2.25)	**< 0.001**
Right ventricle (RV)			
RVEDV (mL)	147 (35.0)	177 (49.1)	0.091
RVEDVI (mL/m^2^)	74.4 (13.1)	93.4 (18.8)	**0.011**
RVESV (mL)	52.6 (18.0)	81.4 (26.0)	**0.001**
RVESVI (mL/m^2^)	26.7 (7.75)	42.7 (11.3)	**0.001**
RV stroke volume (mL)	94.2 (23.5)	95.8 (25.0)	0.855
RVEF (%)	64.4 (7.10)	54.1 (4.31)	**< 0.001**
Tissue characterization			
T1 pre-contrast (ms)	986 (41.0)	923 (30.0)	**< 0.001**
T1ρ (ms)	72.2 (5.86)	65.4 (5.24)	**< 0.001**
T1 post-contrast (ms)	471 (31.1)	476 (38.4)	0.618
ECV (%)	28.1 (3.28)	24.3 (2.24)	**< 0.001**

Values are presented as Mean (Standard Deviation)

p-values < 0.05 are bolded

*CMR* cardiovascular magnetic resonance, *ECV* extracellular volume, *EDV* end diastolic volume, *EDVI* end diastolic volume index, *ESV* end systolic volume, *ESVI* end systolic volume index, *HCM* hypertrophic cardiomyopathy, *LV* left ventricle, *LVEF* left ventricular ejection fraction, *RV* right ventricle, *RVEF* right ventricular ejection fraction

Overall, HCM patients also had higher pre-contrast T1 (986 ms vs. 923 ms; p < 0.001), T1ρ (72.2 ms vs. 65.4 ms; p < 0.001), and ECV (28.1% vs. 24.3%; p < 0.001) compared to controls; T1 post-contrast was not significantly different between HCM patients and controls (p = 0.618). 28 patients (70%) had at least one LGE + + slice.

### Associations between T1, T1ρ, and LGE enhancement in HCM patients

**Fig. 2:**
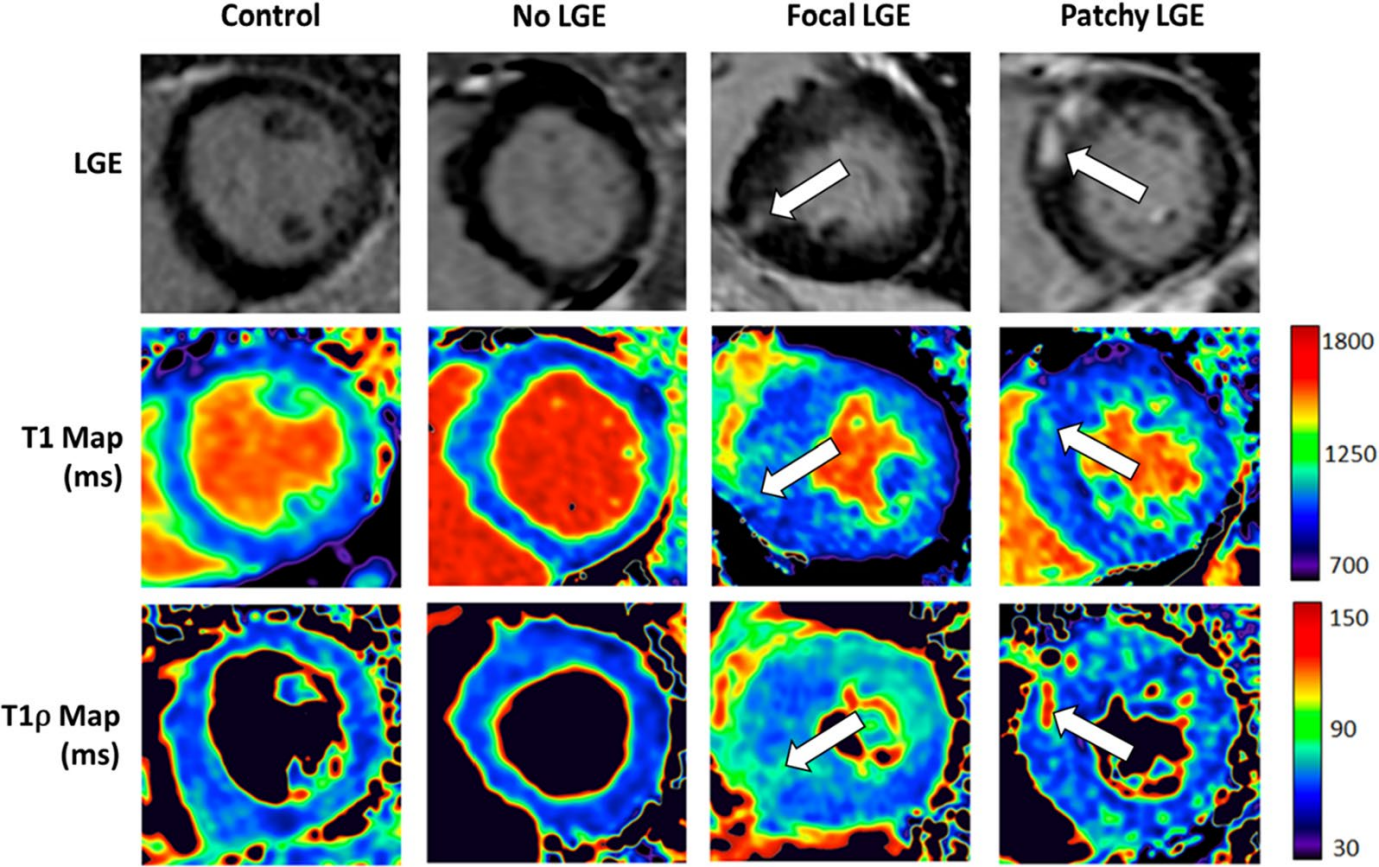
Varying levels of LGE compared to T1⍴ and T1. Short axis LGE, T1, and T1⍴ images are shown for three HCM patients with patchy, focal, and no LGE, and one control patient with no LGE. Areas of elevated T1 and T1⍴ are visually associated with areas of LGE. *LGE* late gadolinium enhancement

**Fig. 3:**
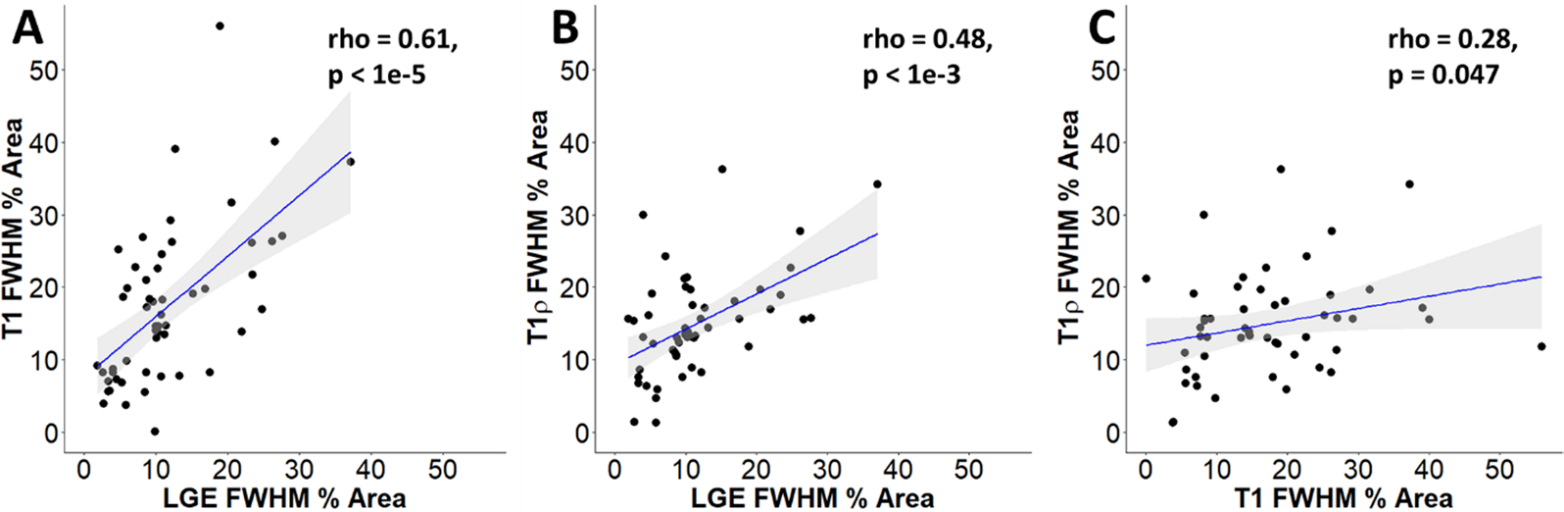
Correlation of T1, T1⍴, and LGE. In LGE-positive slices, we analyzed the correlation of percent area enhancement in **A** T1 versus LGE (Spearman’s rho = 0.61, p < 1e-5), **B** T1⍴ versus LGE (Spearman’s rho = 0.48, p < 1e-3) and **C** T1⍴ versus T1 (Spearman’s rho = 0.28, p = 0.047) images using FWHM thresholding. *FWHM* full width half maximum, *LGE* late gadolinium enhancement

Figure 2 shows T1, T1ρ, and LGE images from three different HCM patients with patchy, focal, and negligible LGE, alongside a control patient with no LGE. Generally, areas of LGE were visibly associated with areas of elevated T1 and T1ρ. In LGE-positive slices, the median area of enhancement within the slice area as assessed by: (1) LGE at FWHM was 10.1% [6.0, 13.7%], (2) native T1 at FWHM was 17.1% [8.3, 22.6%], and (3) T1ρ at FWHM was 14.4% [11.0, 18.1%]. Native T1- and T1ρ-measured enhancement areas were each significantly larger than LGE-measured enhancement area (p < 0.01 for both), while T1ρ- and native T1-measured enhancement area were not significantly different from each other (p = 0.21). Both T1 percent enhancement area (Spearman’s rho = 0.61) and T1ρ percent enhancement area (Spearman’s rho = 0.48) were significantly correlated with LGE percent enhancement area (Fig. 3; p < 0.001 for both). T1 and T1ρ demonstrated a mild correlation (Spearman’s rho = 0.28, p = 0.047).

### Comparisons of T1ρ, native and post-contrast T1, and ECV between LGE categories

**Fig. 4:**
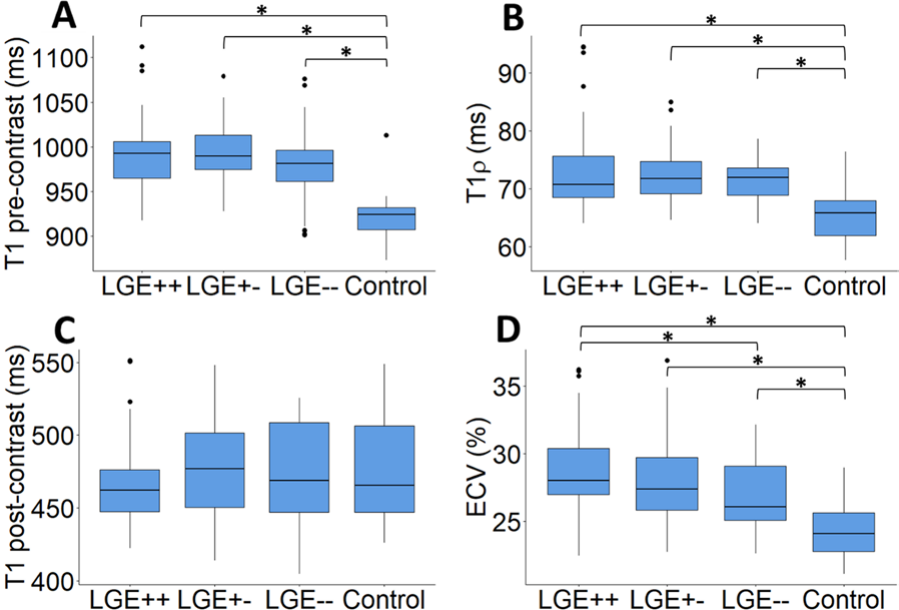
Comparison of myocardial T1⍴, pre-contrast T1, post-contrast T1, and ECV. **A** Average myocardial pre-contrast T1, **B** T1ρ, **C** post-contrast T1, and **D** ECV were measured for HCM patients and controls as indicated. HCM patients were subcategorized by LGE rating: LGE + +, LGE + −, and LGE −−. Kruskal–Wallis test showed statistically significant differences between groups for pre-contrast T1, T1ρ**,** and ECV (p < 0.001 for all). For both T1 and T1ρ, a post-hoc Dunn test showed differences between controls and each LGE category (p < 0.001 for all). For ECV, a post-hoc Dunn test adjusted for multiple comparisons showed differences between controls and each LGE category (p < 0.01 for all), as well as LGE + + and LGE −− (p = 0.01). Statistically significant differences are indicated by * on the bar graphs

To assess whether myocardial tissue characteristics differed by LGE rating, we compared pre-contrast T1, T1ρ, post-contrast T1, and ECV across HCM LGE + +, LGE + −, LGE −−, and control short-axis slices (Fig. 4). For pre-contrast T1, T1ρ, and ECV, Kruskal Wallis test identified differences between groups (p < 0.001 for all); for post-contrast T1, no statistically significant differences were identified. For pre-contrast T1, differences were seen between (1) control and LGE + +, (2) control and LGE + −, and (3) control and LGE −− (p < 0.001 for all). For T1ρ, differences were also seen between (1) control and LGE + +, (2) control and LGE + −, and (3) control and LGE −− (p < 0.001 for all). For ECV, differences were seen between (1) control and LGE + +, (2) control and LGE + −, and (3) control and LGE −− (p < 0.01 for all), as well as (4) LGE + + and LGE −− (p = 0.01).

## Discussion

In our study characterizing the role of endogenous T1ρ imaging in the assessment of patients with HCM, we found that (1) percent area enhancement as measured by T1 and T1ρ at FWHM were moderately correlated with LGE area enhancement, (2) HCM short-axis slices categorized as LGE + +, LGE + −, and LGE −− each demonstrated elevated pre-contrast T1, T1ρ, and ECV compared to controls, and (3) ECV was significantly different between images rated LGE + + compared to LGE −−.

Both T1- and T2-weighted imaging have been used to demonstrate elevations in HCM patient myocardial relaxation times relative to normal patients [5, 25–29]. Cardiac T2 mapping may be sensitive to several different mechanisms of relaxation in vivo. Some of these mechanisms may be considered ‘undesired’ because they suppress ∆T2 between diseased and healthy myocardium. Since each mechanism of relaxation is additive to the overall relaxation rate (i.e., ${R}_{2}={R}_{2,a}+{R}_{2,b}+\dots$, where $a$, $b$, and so on refer to a different relaxation mechanism), eliminating these ‘undesired’ sources of relaxation could increase the difference in the net transverse relaxation. While the ‘unwanted’ contributions to T2 in myocardium are not fully elucidated at present, their effect is to dephase magnetization irreversibly. Potential ‘undesired’ mechanisms of relaxation may include diffusion through background magnetic fields, chemical exchange, among others. By using a sufficiently strong SLk pulse, it is possible to prevent these unwanted mechanisms of relaxation [11]. Using a moderate amplitude (> 400 Hz) SL pulse, we have found that there is a significantly larger ∆T1ρ than ∆T2 in these regions [30]. The net effect of this is an increase in the contrast between normal and diseased myocardium.

Patchy fibrosis occurs in the majority of HCM patients. This is observed primarily as replacement fibrosis, but may also take the form of interstitial fibrosis, which can be imaged and quantified by T1 mapping and subsequent ECV calculation [31, 32]. Most studies of fibrosis in HCM patients have focused on LGE imaging, which allows visualization of replacement fibrosis and has demonstrated associations with adverse outcomes [3]. However, fibrosis accumulates throughout the course of HCM, and additionally, LGE has limited specificity for the prediction of events such as sudden cardiac death and heart failure [8]. It is therefore of both clinical and research interest to investigate new contrast mechanisms such as T1ρ in the HCM population.

To date, only one study has measured T1ρ in human patients with HCM; Wang et al. compared visually-assessed LGE area with 2–6 standard deviation-thresholding of T1ρ in 18 HCM patients, finding high correlation (Pearson’s r ranging from 0.81 to 0.88) of percent fibrosis between these modalities [33]. In our cohort, we found a lower correlation of T1ρ with LGE-assessed enhancement area using Spearman’s rho, which may be due to several reasons. Our cohort is larger with 40 HCM patients and is more heterogenous with both genotype-positive and -negative patients. Additionally, our group applied FWHM thresholding to LGE images, rather than manual measurement of enhancement area, decreasing observer bias. The use of FWHM thresholding therefore increases the robustness of our measurements, allowing for direct comparison in future studies. An additional study of T1ρ in a mouse model of cardiac hypertrophy [34] examined T1ρ at several timepoints after transverse aortic constriction and verified fibrosis ex vivo using Masson’s trichrome staining [34]. Similarly, their findings showed that T1ρ increased over time and was highly correlated with fibrotic areas [34].

Our study brings to light several interesting findings. We show moderate correlations between LGE and T1 and T1ρ-assessed percent enhancement area, and mild correlation between T1 and T1ρ. Variations in the enhancement areas calculated by each method may reflect a physiologic difference in the way that LGE, T1, and T1ρ assess healthy and abnormal tissue. Our results indicate that LGE, T1, and T1ρ may each give different and additive information that one method alone cannot provide, a finding that warrants further study. Additionally, we demonstrate that HCM patients showed elevations in non-contrast quantitative MR measurements (pre-contrast T1 and T1ρ) regardless of LGE status. The significance of T1ρ imaging and its added value will need to be prospectively evaluated.

### Limitations

Several limitations to our study should be acknowledged. Our cohort was small; thus our findings require validation and further investigation in larger groups of patients. Given the low annual cardiovascular event rate in patients with HCM, longer term follow-up will be needed to understand the utility of T1ρ in the assessment of patients with HCM.

## Conclusions

T1 and T1ρ relaxation time moderately correlate with LGE percent enhancement area using FWHM thresholding. Additionally, T1, T1ρ, and ECV distinguish HCM patients from healthy controls, irrespective of whether the patient’s myocardium demonstrated positive LGE, showing potential value as a noninvasive biomarker. Further study is needed to elucidate the role of T1ρ in risk prediction for HCM patients.

## Data Availability

The datasets generated and/or analyzed during the current study are not publicly available due to patient privacy but are available from the corresponding author on reasonable request.

## Abbreviations

ANOVA: One-way analysis of variance
BSA: Body surface area
bSSFP: Balanced steady-state free procession
CMR: Cardiovascular magnetic resonance
ECV: Extracellular volume fraction
EDV: End-diastolic volume
EDVI: End-diastolic volume index
ESC: European Society of Cardiology
ESV: End-systolic volume
TE: Echo time
FWHM: Full width half maximum
GBCAs: Gadolinium-based contrast agents
HCT: Hematocrit
HCM: Hypertrophic cardiomyopathy
IQR: Interquartile range
LGE: Late gadolinium enhancement
LV: Left ventricle/left ventricular
LVEF: Left ventricular ejection fraction
LVEDV: Left ventricular end-diastolic volume
LVEF: Left ventricular ejection fraction
LVESV: Left ventricular end-systolic volume
LVOT: Left ventricular outflow tract
NYHA: New York Heart Association
N_seg_: Number of segments
PSIR: Phase-sensitive inversion recovery
RV: Right ventricle/right ventricular
RVEDV: Right ventricular end-diastolic volume
RVEF: Right ventricular ejection fraction
RVESV: Right ventricular end-systolic volume
SAR: Specific absorption rate
ShMOLLI: Shortened modified Look-Locker inversion recovery
SL: Spin lock
TE: Echo time
TI: Inversion time
TR: Repetition time
TSL: Spin lock time
